# Evaluation of risk factors for acute stroke using combined CTA and MR HR-VWI imaging

**DOI:** 10.3389/fneur.2025.1551682

**Published:** 2025-08-19

**Authors:** Lin Chen, Qian Guo, JiXiu Zhao, Haihua Bao, FanYin Meng, Li Meng

**Affiliations:** ^1^Department of Medical Imaging Center, Affiliated Hospital of Qinghai University, Xining, Qinghai, China; ^2^Department of Radiology, The First Medical Center, Chinese PLA General Hospital, Beijing, China

**Keywords:** carotid artery, periarterial fat, stroke, dangerous patches, inflammatory index

## Abstract

**Objective:**

To investigate the correlation between the changes of peripheral carotid fat density (PFD), the occurrence of acute cerebral ischemia events and the characteristics of different dangerous plaques.

**Methods:**

A retrospective analysis was performed on patients diagnosed with carotid plaque by head and neck CTA in the Affiliated Hospital of Qinghai University from January 2021 to March 2023. All patients received head magnetic plain scan, DWI and high resolution vascular wall imaging (MR HR-VWI). According to DWI images, the patients were divided into acute cerebral infarction group and non-acute cerebral infarction group, and the clinical data, CT features and PFD differences between the two groups were compared. Logistic regression analysis was used to adjust for confounding factors and calculate OR values. ROC curves were used to evaluate the predictive efficacy of symptomatic PFD, contralateral PFD and differential PFD for symptomatic and non-symptomatic carotid plaque. According to the CTA and MR HR-VWI, the patients were further divided into groups (calcification and non-calcification, ulcer and non-ulcer, intra-plaque bleeding and non-plaque bleeding, thin or broken fibrous cap and non-thin or broken fibrous cap, large lipid core and non-large lipid core). Multifactor linear regression equation was used to compare the differences of symptomatic side PFD, contralateral PFD and differential PFD among different groups, and to analyze the correlation between PFD and different plaque components. A retrospective analysis was conducted on patients diagnosed with carotid atherosclerotic plaques via head and neck CTA at Qinghai University Affiliated Hospital between January 2021 and March 2023. All patients underwent non-contrast brain MRI with diffusion-weighted imaging (DWI) and high-resolution vessel wall imaging (MR HR-VWI).

**Results:**

(1) Clinical and Imaging Features: The acute stroke group demonstrated significantly elevated systolic (159.2 ± 28.35 vs. 143 ± 25.54 mmHg, *p* = 0.019) and diastolic blood pressures (93.67 ± 15.75 vs. 84.60 ± 13.21 mmHg, *p* = 0.016) compared to the non-acute group. Additionally, the acute stroke group exhibited greater plaque thickness (4.4 ± 1.4 vs. 2.9 ± 0.9 mm, *p* < 0.001), higher prevalence of severe stenosis (45.8% vs. 4.0%, *p* = 0.001), and more frequent ulcerated or irregular plaque surfaces (29.2% vs. 8.0%, *p* = 0.038). (2)Predictive Efficacy of PFD: In predictive analyses, symptomatic-side PFD showed superior performance in identifying acute ischemic events (AUC = 0.762, 95% CI: 0.653–0.870) compared to contralateral PFD (AUC = 0.672) and ΔPFD (AUC = 0.660). (3)Association with Plaque Components: Multivariate regression analysis revealed significant associations between symptomatic-side PFD and key plaque characteristics: intraplaque hemorrhage (IPH; *β* = 0.367, *p* < 0.001), lipid-rich necrotic core (LRNC; *β* = 0.190, *p* = 0.046), and plaque thickness (*β* = 0.225, *p* = 0.027). Notably, IPH exhibited the strongest correlation with PFD values among all evaluated components.

**Conclusion:**

Carotid perivascular fat density (PFD) can be used as a potential imaging marker to evaluate the characteristics of local vascular inflammation and high-risk plaques, providing a new direction for the early diagnosis and targeted therapy of acute cerebral ischemic events.

## 1 Background

Stroke is a significant global health issue, characterized by high incidence, mortality, and disability rates, with a rising prevalence and a trend toward younger age groups (1–3). In China, stroke is one of the main causes of death, of which ischemic stroke accounts for the majority, and about 1/4 ischemic stroke is closely related to carotid atherosclerosis (4).

Carotid atherosclerotic plaque is the key pathological basis of stroke. Early studies focused on the degree of arterial lumen stenosis, but in recent years, studies have gradually turned to the analysis of the composition and characteristics of vulnerable plaques. Vulnerable plaques are mainly manifested as thin or ruptured fiber caps, large lipid cores, intra-plaque bleeding (IPH), plaque ulceration, inflammatory response, and neovascularization (5). The presence of these high-risk features greatly increases the likelihood of plaque rupture and stroke. Therefore, accurate identification and evaluation of these dangerous plaque components is of great clinical significance for the prevention of stroke.

Among them, inflammation plays a key role in the formation and progression of vulnerable plaques. However, compared with other plaque features, inflammation has become a research and clinical diagnosis challenge because it is difficult to be visually detected by traditional imaging methods. In recent years, detection of fluorine-18 markers based on positron emission tomography (PET-CT) has been used to assess intravascular inflammation, but its high cost and complexity have limited widespread application.

With advances in imaging technology, the coronary perivascular fat attenuation index (FAD) has emerged as a novel non-invasive biomarker for evaluating vascular inflammation (6, 7). Studies have shown that perivascular adipose tissue (PVAT) exhibits paracrine and bidirectional interactions with the vascular wall (8). During vascular inflammation, the physiological state of PVAT changes, resulting in reduced fat content and increased CT attenuation values.

Research on carotid perivascular fat density (PFD) remains limited. Some studies suggest a close association between PFD and vulnerable plaque characteristics. For example, Zhang et al. reported significantly higher PFD in patients with IPH compared to those without IPH (9). Another study indicated that PFD was elevated in patients with acute ischemic stroke, with higher values observed on the infarction side compared to the contralateral side, suggesting that changes in PFD may reflect active local inflammation (10). Despite these findings, the relationship between PFD and specific vulnerable plaque components has not been thoroughly explored, emphasizing the need for further investigation in this area.

This study aims to investigate the association between perivascular fat density (PFD) of the carotid artery and different vulnerable plaque characteristics by evaluating PFD. Its significance lies in providing a novel noninvasive imaging biomarker for early stroke warning and personalized treatment. Conventional imaging modalities are limited in directly assessing plaque inflammatory activity, whereas PFD quantifies density changes in perivascular adipose tissue to indirectly reflect the degree of local inflammation. The findings are expected to offer new insights into the in-depth research on the pathological mechanisms of carotid atherosclerosis and the development of related diagnostic and therapeutic strategies.

## 2 Materials and methods

### 2.1 General information

This study employed a retrospective analysis of data from patients diagnosed with carotid atherosclerotic plaques via head and neck CTA at Qinghai University Affiliated Hospital between January 2021 and March 2023. All patients underwent non-contrast brain MRI, diffusion-weighted imaging (DWI), and high-resolution vessel wall imaging (MR HR-VWI) after hospital admission. Ethical approval was obtained from the hospital’s ethics committee, and since imaging examinations were part of routine clinical diagnostics, separate informed consent was not required.

**Inclusion Criteri**: Patients were included if they met both of the following conditions: (1) the time interval between head and neck CTA, MR HR-VWI, and non-contrast brain MRI examinations did not exceed 2 weeks; and (2) demonstrated carotid plaque thickness exceeding 1.5 mm on imaging.

**Exclusion Criteria**: Participants were excluded for any of the following reasons: (1) presence of non-atherosclerotic vascular pathologies including aneurysms or vasculitis; (2) acquisition of poor-quality CT or MRI images precluding accurate analysis; (3) previous history of carotid interventions such as stenting or endarterectomy; or (4) advanced age or physical conditions contraindicating imaging procedures.

**Data Collection**: Comprehensive clinical and laboratory data were systematically collected, encompassing demographic characteristics (gender, age), medical history (hypertension, diabetes, coronary artery disease), lifestyle factors (smoking status, alcohol use), and lipid profiles (total cholesterol, triglycerides, high-density lipoprotein, low-density lipoprotein). All clinical data were obtained within a two-week window surrounding the imaging studies to ensure temporal relevance.

**Definitions**: Symptomatic carotid artery: The carotid artery on the same side as an acute ischemic lesion in the internal carotid artery territory, as identified on DWI, or the artery associated with neurological symptoms (9).

**Contralateral carotid artery**: The carotid artery on the opposite side of the symptomatic carotid artery.

### 2.2 Imaging protocols

#### 2.2.1 CTA imaging protocol

All examinations were performed using a 256-slice Revolution CT scanner (GE Healthcare) in spiral scanning mode, with coverage extending from the aortic arch to the cranial vault. A bolus of 60–80 mL iodinated contrast agent (Omnipaque-350; GE Healthcare) was administered intravenously at 4 mL/s, followed by a 40 mL saline flush using a power injector, with scan initiation triggered automatically 5 s after reaching an attenuation threshold of 100 HU in the aortic arch. Standard acquisition parameters included: tube voltage 100 kV, pitch 1.0, reconstruction slice thickness 0.5 mm, slice spacing 0.5 mm, and rotation time 350 ms. The effective dose (ED) was calculated as ED = DLP × K, where K represents the radiation conversion factor (0.0023 mSv·mGy^−1^·cm^−1^) for adult head and neck examinations.

#### 2.2.2 High-resolution HR-VWI imaging protocol

All MR examinations were performed using 3.0 T scanners (Philips Healthcare Discovery 750 W and GE Healthcare systems). The standardized imaging protocol comprised two-dimensional time-of-flight (TOF), coronal three-dimensional T1-weighted imaging (CORONAL-3D T1WI), axial T1WI, axial T2WI, contrast-enhanced magnetic resonance angiography (CE-MRA), and contrast-enhanced three-dimensional T1-weighted imaging (CE-3D T1WI) sequences. For contrast-enhanced studies, gadopentetate dimeglumine (Gd-DTPA) was administered intravenously at a dose of 0.2 mmol/kg body weight, injected at 2.5 mL/s using a high-pressure injector (Ulrich, Germany).

#### 2.2.3 Brain MRI + DWI

All brain MRI examinations were conducted using a Siemens Prisma 3.0 T scanner following standard diffusion-weighted imaging (DWI) protocols. The imaging protocol included four essential sequences: (1) axial T1-weighted imaging (T1WI) with TR/TE = 150/2.5 ms, (2) axial T2-weighted imaging (T2WI) with TR/TE = 5000/117 ms, (3) T2 FLAIR with TR/TE = 8000/81 ms, and (4) diffusion-weighted imaging (DWI) with b-value = 1,000 s/mm^2^ and TR/TE = 3230/65 ms. All sequences shared consistent geometric parameters: field of view (FOV) 230 × 160 mm and slice thickness 5.0 mm.

### 2.3 Carotid perivascular fat density measurement

The PFD measurement method was based on established coronary artery fat measurement techniques, combined with semi-automated segmentation to determine perivascular fat density along the vascular narrowing region.

**Figure 1:**
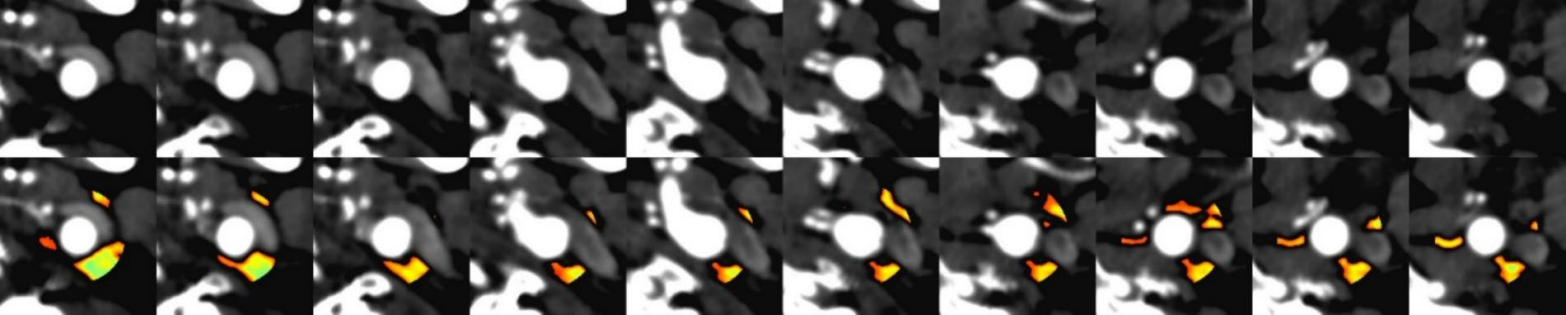
Comparison of CT angiography (CTA) imaging and automated perivascular fat density extraction: **(A)** Upper row: Conventional CTA cross-sectional images. **(B)** Lower row: Corresponding images with automated fat density extraction, where color-coded regions indicate perivascular adipose tissue (PVAT) distribution.

**Definition**: PFD was quantified by adjusting the technical parameters on the perivascular fat attenuation histogram within the range of −190 to −30 HU, with all fat density measurements reported in Hounsfield Units (HU), See Figure 1.

**Procedure**: Quantitative image analysis was performed using dedicated vascular imaging software (Shukun, version 6.21.730.3) for automated delineation of regions of interest (ROIs) and subsequent computational analysis. The perivascular fat density (PFD) difference (ΔPFD) was calculated by subtracting contralateral-side PFD values from symptomatic-side PFD values (ΔPFD = PFDsymptomatic–PFDcontralateral), with all measurements generated through the software’s automated quantification algorithm.

### 2.4 Image interpretation

All images were independently reviewed by two radiologists with over 5 years of experience in carotid plaque analysis. In case of disagreement, a consensus was reached through discussion.

Stenosis Degree: Evaluated according to the NASCET (North American Symptomatic Carotid Endarterectomy Trial) standards (10).

**Figure 2:**
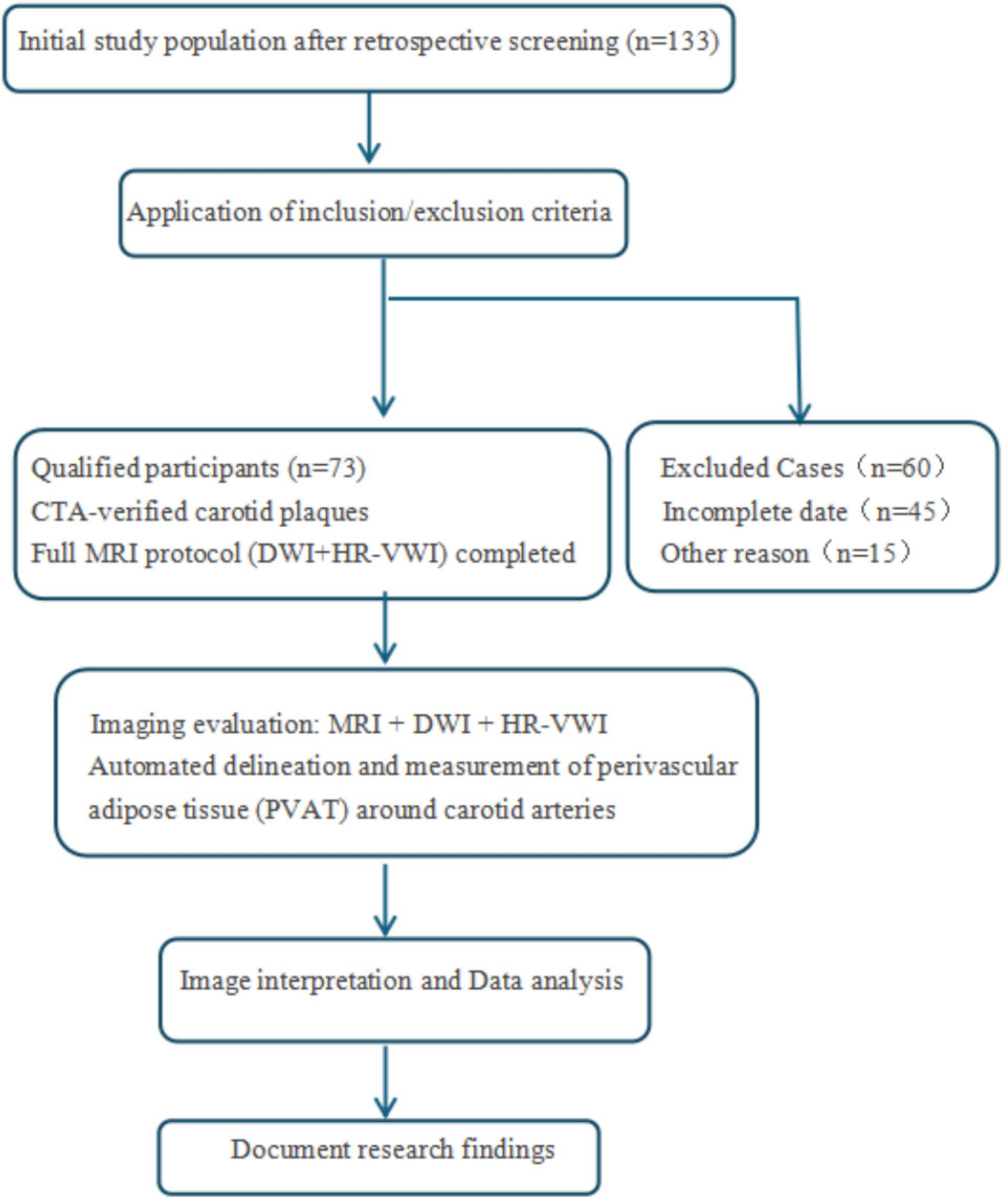
Flowchart.

The hallmark features of vulnerable plaques include: intraplaque hemorrhage (IPH), a thin or disrupted fibrous cap (<65 μm), an extensive lipid-rich necrotic core (>40% of plaque volume), prominent inflammatory cell infiltration (predominantly macrophages and T-lymphocytes), and pathological calcification patterns (particularly microcalcifications; Figure 2).

### 2.5 Statistical methods

Data analysis was performed using SPSS 25.0 software, with the following methods:

Continuous variables were first tested for normality using the Shapiro–Wilk test. Normally distributed data were presented as mean ± standard deviation (mean±SD), with between-group comparisons performed using independent samples t-tests. Non-normally distributed data were expressed as median (interquartile range), with between-group comparisons analyzed using the Mann–Whitney U test. Paired data comparisons employed paired t-tests, For non-normally distributed paired data, the Wilcoxon signed-rank test will be used.

Categorical variables were presented as counts (percentages), with between-group differences evaluated using chi-square tests.

The following regression models were applied: (1) binary logistic regression to analyze the relationships between symptomatic-side PFD, contralateral-side PFD, ΔPFD and acute ischemic events; (2) multiple linear regression to examine associations between symptomatic-side PFD (dependent variable) and plaque characteristics (independent variables). The statistical significance level was set at *p* < 0.05.

Diagnostic performance of PFD measures was evaluated through receiver operating characteristic (ROC) curve analysis, calculating the area under the curve (AUC) and determining optimal cutoff values.

## 3 Results

### 3.1 General characteristics comparison

A total of 73 patients were included in the study, with an average age of 61 ± 12.5 years. Among them, 51 were male (69.9%) and 58 were of Han ethnicity (79.5%). The prevalence of hypertension, dyslipidemia, diabetes, and smoking was 69.9, 45.2, 27.4, and 41.1%, respectively (Table 1).

**Table 1:** Clinical characteristics of the study population.

Characteristic	Value (Mean ± SD) or n (%)
Age (years)	61 ± 12.5
Gender (Male)	51 (69.9)
Ethnicity (Han)	58 (79.5)
History of Hypertension	51 (69.9)
Systolic Blood Pressure (mmHg)	153.68 ± 28.33
Diastolic Blood Pressure (mmHg)	90.56 ± 15.45
History of Dyslipidemia	33 (45.2)
Total Cholesterol (mmol/L)	4.06 ± 1.15
Triglycerides (mmol/L)	1.64 ± 0.87
High-Density Lipoprotein (HDL; mmol/L)	0.96 ± 0.24
Low-Density Lipoprotein (LDL; mmol/L)	2.53 ± 0.92
History of Diabetes	20 (27.4)
History of Coronary Artery Disease	2 (2.7)
History of Smoking	30 (41.1)
History of Alcohol Consumption	32 (43.8)

### 3.2 Comparison of clinical, CTA features, and carotid PFD between acute and non-acute ischemic stroke groups

Among the 73 cases, there were 48 patients with acute cerebral infarction and 25 with non-acute cerebral infarction. The non-acute ischemic stroke group, the acute ischemic stroke group exhibited:

The acute ischemic stroke group demonstrated significantly elevated systolic blood pressure (159.2 ± 28.35 vs. 143 ± 25.54 mmHg, *p* = 0.019) and diastolic blood pressure (93.67 ± 15.75 vs. 84.60 ± 13.21 mmHg, *p* = 0.016) compared to the non-acute group. Additionally, plaque thickness was markedly greater in the acute group (4.4 ± 1.4 vs. 2.9 ± 0.9 mm, *p* < 0.001), with a higher prevalence of severe stenosis (45.8% vs. 4.0%, *p* = 0.001) and plaque surface ulceration/irregularity (29.2% vs. 8.0%, *p* = 0.038). However, no significant differences were observed in plaque length (*p* = 0.067) or calcification (*p* = 0.516) between the two groups (Table 2).

**Table 2:** Comparison of clinical, CTA features, and carotid PFD between acute and non-acute ischemic stroke patients.

Parameter	Acute ischemic stroke (*n* = 48)	Non-acute ischemic stroke (*n* = 25)	*p*
Age (years)	63.23 ± 13.08	58.52 ± 10.79	0.127
Gender (Male)	36 (75.0)	15 (60.0)	0.185
Ethnicity (Han)	39 (81.3)	19 (76)	0.598
History of Hypertension	35 (72.9)	15 (60.0)	0.194
Systolic Blood Pressure (mmHg)	159.2 ± 28.35	143 ± 25.54	0.019
Diastolic Blood Pressure (mmHg)	93.67 ± 15.75	84.60 ± 13.21	0.016
History of Dyslipidemia	29 (60.4)	14 (56.0)	0.453
Total Cholesterol (mmol/L)	4.11 ± 1.16	3.96 ± 1.56	0.589
Triglycerides (mmol/L)	1.67 ± 0.89	1.61 ± 0.85	0.569
High-Density Lipoprotein (HDL; mmol/L)	0.96 ± 0.23	0.97 ± 0.27	0.872
Low-Density Lipoprotein (LDL; mmol/L)	2.58 ± 0.95	2.42 ± 0.87	0.478
History of Diabetes	13 (27.1)	7 (28.0)	0.934
History of Coronary Artery Disease	2 (4.2)	0 (0.0)	0.301
History of Smoking	18 (37.5)	12 (48.0)	0.387
History of Alcohol Consumption	19 (39.6)	13 (52.0)	0.222
History of Antihypertensive Medication Use	26 (54.17)	7 (28.0)	0.029
History of Antiplatelet Medication Use	3 (6.3)	2 (8.0)	0.562
History of Statin Use	4 (8.3)	2 (8.0)	0.666
Symptomatic-side PFD (HU)	−53.63 ± 11.04	−65.40 ± 10.80	<0.001
Contralateral-side PFD (HU)	−77.16 ± 10.64	−83.08 ± 9.94	0.024
ΔPFD (HU)	23.53 ± 10.71	17.68 ± 7.28	0.017
Plaque Thickness (mm)	4.4 ± 1.4	2.9 ± 0.9	<0.001
Plaque Length (mm)	10.1 ± 5.3	7.8 ± 4.3	0.067
Degree of Stenosis (Severe)	22 (45.8)	1 (4.0)	0.001
Ulceration or Irregular Plaque Surface	14 (29.2)	2 (8.0)	0.038
Calcification	33 (68.8)	19 (76)	0.516

ΔPFD (HU), Symptomatic-side PFD–Contralateral-side PFD.

In patients with acute ischemic stroke, the symptomatic-side PFD, contralateral carotid PFD, and △PFD (HU) were (−53.63 ± 11.04) HU, (−77.16 ± 10.64) HU, and (23.53 ± 10.71) HU, respectively. In non-acute ischemic stroke patients, the symptomatic-side PFD, contralateral carotid PFD, and △PFD (HU) were (−65.40 ± 10.80) HU, (−83.08 ± 9.94) HU, and (17.68 ± 7.28) HU, respectively. The differences between the two groups were statistically significant (*p* < 0.05; see Table 2).

### 3.3 Perivascular fat density (PFD) comparison of symptomatic versus contralateral carotid arteries in all patients

Within the same patient, symptomatic-side PFD (−57.66 ± 12.25 HU) was significantly higher than contralateral-side PFD (−79.19 ± 10.72 HU), with an average ΔPFD of 21.53 ± 10.02 HU (*p* < 0.001; Table 3).

**Table 3:** Comparison of symptomatic-side PFD and contralateral-side PFD in the same patient.

Symptomatic-side PFD (HU)	Contralateral-side PFD (HU)	Difference (ΔPFD; HU)	Number of cases	*t*	*p*
−57.66 ± 12.25	−79.19 ± 10.72	21.53 ± 10.02	73	18.36	<0.001

### 3.4 Comparison of PFD at different degrees of stenosis and PFD of different plaque compositions

In the study, 50 patients (68.5%) had mild-to-moderate stenosis, and 23 patients (31.5%) had severe stenosis. The symptomatic-side PFD was higher in the severe stenosis group (−50.66 ± 10.36 HU) than in the mild-to-moderate stenosis group (−60.88 ± 11.78 HU, *p* = 0.001; Table 4).

**Table 4:** Comparison of PFD at different degrees of stenosis and PFD of different plaque compositions.

Plaque characteristics	Mild-to-moderate stenosis (*n* = 50)	Severe stenosis (*n* = 23)	*p*	Ulcerated group (*n* = 16)	Non-ulcerated group (*n* = 57)	*p*
Symptomatic-side PFD (HU)	−60.88 ± 11.78	−50.66 ± 10.36	0.001	−56.66 ± 9.91	−57.95 ± 12.89	0.713
Contralateral-side PFD (HU)	−80.39 ± 10.71	−76.59 ± 10.50	0.161	−75.76 ± 10.03	−80.15 ± 10.79	0.148
ΔPFD (HU)	19.50 ± 9.38	25.93 ± 10.15	0.010	19.09 ± 11.11	22.21 ± 9.69	0.276
	Calcified group (*n* = 50)	Non-calcified group (*n* = 23)	*p*	TRFC group (*n* = 16)	Non-TRFC group (*n* = 57)	*p*
Symptomatic-side PFD (HU)	−58.44 ± 12	−55.73 ± 12.93	0.395	−51.61 ± 9.95	−59.50 ± 12.36	0.019
Contralateral-side PFD (HU)	−80.23 ± 10.75	−76.61 ± 10.46	0.194	−74.62 ± 8.12	−80.58 ± 11.08	0.044
ΔPFD (HU)	21.79 ± 9.43	20.88 ± 11.57	0.730	−23.01 ± 9.27	21.08 ± 12.36	0.489
	LRNC group (*n* = 22)	Non-LRNC group (*n* = 51)	*p*	IPH group (*n* = 15)	Non-IPH group (*n* = 58)	*p*
Symptomatic-side PFD (HU)	−50.84 ± 11.03	−60.42 ± 11.72	0.002	−43.82 ± 7.58	−61.24 ± 10.58	<0.001
Contralateral-side PFD (HU)	−75.35 ± 8.84	−80.74 ± 11.09	0.051	−69.35 ± 8.05	−81.74 ± 9.86	<0.001
ΔPFD (HU)	24.51 ± 12.56	20.32 ± 8.64	0.172	25.53 ± 12.93	20.49 ± 8.96	0.172

Based on plaque composition, all patients were divided into calcified and non-calcified groups, IPH and non-IPH groups, TRFC and non-TRFC groups, and LRNC and non-LRNC groups. Statistically significant differences were found between the IPH and non-IPH groups, as well as between the TRNC and non-TRNC groups (*p* < 0.05). However, no statistical differences were observed between the calcified and non-calcified groups, or between the ulcerated and non-ulcerated groups (Table 4).

### 3.5 Binary logistic regression analysis of risk factors for acute ischemic stroke

Symptomatic-side PFD, contralateral PFD, and ΔPFD were all significantly associated with acute ischemic events (*p* < 0.05). After adjusting for systolic blood pressure, diastolic blood pressure, antihypertensive medication use, plaque thickness, and stenosis degree, symptomatic-side PFD remained predictive (OR = 0.919, *p* = 0.044; Table 5).

**Table 5:** PFD and acute ischemic event models.

Variables	Model 1		Model 2		Model 3	
	OR(95%CI)	*p*	OR(95%CI)	*p*	OR(95%CI)	*p*
Symptomatic-side PFD	0.895(0.840–0.954)	0.001	0.889(0.831–952)	0.001	0.919(0.846–0.998)	0.044
Contralateral-side PFD	0.947(0.902–995)	0.030	0.950(0.902–1.001)	0.056	0.957(0.897–1.021)	0.185
ΔPFD	0.933(0.879–990)	0.023	0.920(0.862–0.981)	0.012	0.967(0.897–1.043)	0.387

Model 1: Regression analysis of PFD only. Model 2: Adjusted for systolic blood pressure, diastolic blood pressure, and antihypertensive medication use. Model 3: Adjusted for systolic blood pressure, diastolic blood pressure, antihypertensive medication use, plaque thickness, and degree of stenosis.

#### 3.5.1 ROC curve analysis of symptomatic-side PFD, contralateral-side PFD, and ΔPFD in predicting acute ischemic stroke

**Figure 3:**
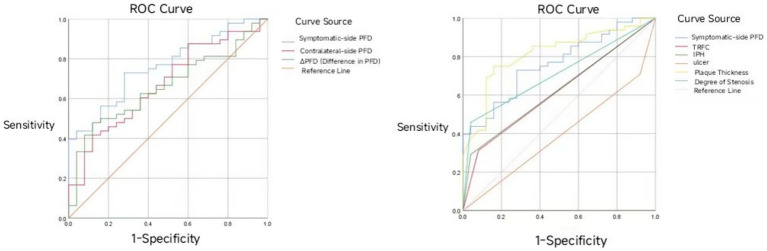
ROC curve of symptomatic-side PFD, contralateral-side PFD, and ΔPFD in predicting acute ischemic stroke.

Symptomatic-side PFD, contralateral-side PFD, and ΔPFD all demonstrated certain predictive value for acute ischemic stroke (*p* < 0.05). The predictive efficacy was ranked as follows: symptomatic-side PFD > contralateral-side PFD > ΔPFD. Among these, symptomatic-side PFD had the best predictive ability for acute ischemic stroke, with an area under the curve (AUC) of 0.762 (95% CI: 0.653–0.870; Table 6), and the optimal cutoff value is indicated in Figure 3.

**Table 6:** Predictive values of symptomatic-side PFD, contralateral PFD, and △PFD for acute cerebral infarction.

Variables	Cut-off value	AUC	95% CI	Sensitivity (SE)	Specificity (SP)
Symptomatic-side PFD	0.449	0.762	0.653 ~ 0.870	0.729	0.64
Contralateral PFD	0.297	0.672	0.545 ~ 0.799	0.417	0.88
△PFD	0.359	0.660	0.535 ~ 0.785	0.419	0.88

Explanation: AUC, Area Under the Curve; 95% CI, 95% Confidence Interval; SE (Sensitivity), The ability to correctly identify positive cases (True Positive Rate); SP (Specificity), The ability to correctly identify negative cases (True Negative Rate).

### 3.6 Multiple linear regression analysis of the relationship between symptomatic-side PFD and different plaque components

A multiple linear regression analysis was performed with symptomatic-side PFD as the dependent variable, and IPH, TRFC, LRNC, plaque thickness, and stenosis degree as independent variables. The results showed that IPH, LRNC, and plaque thickness were significantly associated with symptomatic-side PFD (*p* < 0.05), with IPH being the most significant (*p* < 0.001; Table 7).

**Table 7:** Multivariate linear regression analysis of the relationship between symptomatic side PFD and different plaque components.

Variable	B	*β*	*t*	*p*	95% CI	
					Lower limit	Upper limit
Plaque thickness	19.035	0.225	2.259	0.027	2.215	35.854
TRFC (Thin, Ruptured Fibrous Cap)	4.708	0.164	1.817	0.074	−0.463	9.879
IPH (Intraplaque Hemorrhage)	11.054	0.367	3.710	<0.001	5.107	17.001
LRNC (Lipohyalinosis)	5.102	0.190	2.034	0.046	0.095	10.109
Degree of stenosis	4.385	0.167	1.731	0.088	−0.672	9.441

## 4 Discussion

This study investigated the association between perivascular fat density (PFD) and acute cerebral infarction events as well as vulnerable plaque characteristics. The results demonstrated that patients in the acute infarction group exhibited significantly higher PFD values in the symptomatic side, contralateral side, and ΔPFD compared to the non-acute infarction group (p < 0.05), consistent with recent findings (11, 27), further confirming that elevated perivascular PFD serves as an important predictor for cardiovascular risk. ROC curve analysis revealed that PFD had good predictive performance for acute cerebral ischemic events, with the symptomatic-side PFD showing optimal predictive efficacy (AUC 0.762, 95% CI 0.653–0.870), indicating potential clinical utility. As a noninvasive imaging biomarker, PFD may play a significant role in assessing vascular inflammation and predicting high-risk plaques, serving as a novel imaging marker for vascular inflammatory activity and vulnerable plaques. This study provides preliminary evidence supporting its application in acute cerebral ischemic events. Future research should focus on: establishing standardized PFD measurement protocols; validating its predictive value through multicenter prospective cohorts; and elucidating the mechanistic relationship between PFD and molecular inflammatory markers - ultimately facilitating clinical translation of this noninvasive parameter for early identification and targeted intervention in high-risk populations.

Additionally, the study found that PFD is closely related to plaque thickness, intraplaque hemorrhage (IPH), and lipid-rich necrotic core (LRNC). Among these, IPH had the most significant effect on PFD (*p* < 0.001), which aligns with findings from Miao Yu and Zhang S (9, 12). This suggests that PFD, as an alternative imaging biomarker for local vascular inflammation and vulnerable plaques, can provide a convenient method for diagnosing and predicting high-risk ischemic events, while also offering new therapeutic targets for clinical interventions (13).

The core mechanism of atherosclerosis is the inflammatory response. The initial injury to the endothelium leads to the accumulation of macrophages and monocytes, forming different types of plaques that subsequently induce lumen narrowing. Excessive narrowing can increase distal circulation pressure and reduce metabolism, resulting in ischemia and hypoxia in brain tissue. The rupture of vulnerable plaques can also cause distal infarction.

Recent studies have suggested a bidirectional secretion effect between the vascular wall and the surrounding adipose tissue (perivascular adipose tissue, PVAT), which is considered a key protective factor in the cardiovascular system (14, 15). Inflammation can alter the composition of PVAT from a lipid phase to a water phase, increasing its CT density in the inflammatory vascular areas (16). At the same time, inflammatory factors secreted by PVAT can recruit more inflammatory cells, further exacerbating local inflammation (15). The increase in PVAT density is also independently associated with the progression of the lipid components of coronary atherosclerotic plaques (17), providing theoretical support for its use as an imaging marker.

The composition and stability of carotid plaques directly influence the risk of systemic cardiovascular events (18–20). For example, strong evidence shows that IPH and plaque ulceration are associated with an increased risk of ipsilateral ischemic stroke (19), and the size of LRNC is highly correlated with the risk of ischemic stroke (18). This study found that patients with vulnerable plaque features such as IPH, TRFC (thin or ruptured fibrous cap), and LRNC had significantly higher PFD, further supporting the potential of PFD as a predictive factor for high-risk plaques, consistent with findings from Yu et al. (12).

However, in our study, there was no statistical difference in PFD between calcified and non-calcified plaques. We speculate that this may be related to the grouping, as mixed plaques contain both calcification and other components such as IPH or TRFC. Simply classifying them as calcified and non-calcified could affect the accuracy of the results. Additionally, we believe this may be due to the small sample size and selection bias, which may be addressed in future studies by expanding the sample size and minimizing such biases. Nevertheless, the PFD value in the calcified group was slightly lower than in the non-calcified group, which aligns with previous studies where calcification was considered a protective factor (21, 22). Plaque calcification is closely related to plaque stability and inversely correlated with inflammation (21, 23, 24). Moreover, no significant difference in PFD was found between ulcerated and non-ulcerated plaques, consistent with Zhang S’s findings (9), suggesting that the impact of ulceration on PFD might be minor.

Previous studies have indicated that an increase in carotid plaque thickness is closely related to symptomatic disease status (25), with inflammatory stimuli accelerating plaque growth, leading to lumen narrowing and thickening of the vessel wall. In a cross-sectional study involving 1,072 patients with brain ischemia, plaque thickness was found to be a better predictor of ischemic symptoms than stenosis degree (26). Another study pointed out that for every 1 mm increase in soft plaque thickness, the risk of ipsilateral ischemic events increases 3.7 times (95% CI: 1.9–7.2) (22). ROC curve analysis further supports plaque thickness as an important risk predictor, with an AUC of 0.88, a best cutoff value of 2.2 mm, sensitivity of 85%, and specificity of 83%.

### 4.1 Limitations

Although this study preliminarily validated the value of PFD in assessing vascular local inflammation and predicting acute cerebral ischemic events, there are still some limitations:

1. Retrospective design: The occurrence of future ischemic events cannot be predicted and needs to be verified by prospective studies with larger sample sizes and longer follow-up periods.
2. Small sample size: There may be bias in the group results, especially in the comparison of calcified and non-calcified plaques.
3. Limitations of measurement methods: The measurement of PFD may be affected by differences in placement of ROI. This study used a coronary approach based. Although this study provides preliminary evidence for the value of PFD in assessing local vascular inflammation and predicting acute cerebral ischemic events, it has several limitations.
